# Analysis of new treatments proposed for malignant pleural mesothelioma raises concerns about the conduction of clinical trials in oncology

**DOI:** 10.1186/s12967-022-03744-6

**Published:** 2022-12-13

**Authors:** Tomer Meirson, Valerio Nardone, Francesca Pentimalli, Gal Markel, David Bomze, Maria D’Apolito, Pierpaolo Correale, Antonio Giordano, Luigi Pirtoli, Camillo Porta, Steven G Gray, Luciano Mutti

**Affiliations:** 1 Davidoff Cancer Center, Rabin Medical Center-Beilinson Hospital, 49100 Petah Tikva, Israel; 2 Department of Precision Oncology, University Hospital of Campania L. Vanvitelli, Naples, Italy; 3 Dipartimento di Medicina e Chirurgia, Libera Università Mediterranea “Giuseppe Degennaro”, Bari, Italy; 4 Sackler Faculty of Medicine, Tel Aviv University, Tel Aviv, Israel; 5 Unit of Medical Oncology, Oncology Department, Grand Metropolitan Hospital Bianchi Melacrino Morelli, Reggio Calabria, Italy; 6 Sbarro Institute for Cancer Research and Molecular Medicine, Center for Biotechnology, College of Science and Technology, Temple University, Philadelphia, PA USA; 7 Department of Medical Biotechnologies, University of Siena, Siena, Italy; 8 Interdisciplinary Department of Medicine, University of Bari “Aldo Moro” and A.O.U. Consorziale Policlinico di Bari, Bari, Italy; 9 Thoracic Oncology Research Group, Trinity St James’s Cancer Institute, St James’s Hospital, Dublin, Ireland; 10 Department of Biotechnological and Applied Clinical Sciences, University of L’Aquila, Via Vetoio, Coppito 2, 67100 L’Aquila, Italy

## Abstract

In this commentary, using existing clinical trial data and FDA approvals we propose that there is currently a critical need for an appropriate balancing between the financial impact of new cancer drugs and their actual benefit for patients. By adopting “pleural mesothelioma” as our clinical model we summarize the most relevant pertinent and available literature on this topic, and use an analysis of the reliability of the trials submitted for registration and/or recently published as a case in point to raise concerns with respect to appropriate trial design, biomarker based stratification and to highlight the ongoing need for balancing the benefit/cost ratio for both patients and healthcare providers.

## Background

Over the course of the last few years the design and analysis of clinical trials have come under scrutiny mainly because of the increasing number of new treatments approved coupled with the need to understand their actual clinical and economic impact.

We have noted an increasing number of registrations for a relatively limited number of costly drugs/classes of drugs for a broad range of different human tumors. Therefore, a potential discrepancy has arisen in that there is a risk that the preclinical rationale and the actual clinical benefit/economic ratio leading to these registrations have not been sufficiently scrutinized.

In this regard we and others have cautioned that given the increasing cost of these drugs together with their safety profile it is recommended their approval in clinical practice should only be granted only when they show a proven impact both clinically and economically [1–3].

Despite several red flags that have been raised (e.g. [4]), FDA approved treatments have boomed during the last year which may lead to significantly increased costs for the health systems worldwide.

There is no doubt that such treatments in this era of precision oncology have the potential to greatly improve the clinical outcomes of distinct subsets of patients [5]. We believe there are limitations to the current approvals with respect to the following: (a) the vast majority of these approvals appear to have been inspired by a “one-size fits-all” approach rather to a patient / disease tailored treatment [6]; (b) the pricing of these new targeted therapies did not differ regardless of if they achieved a surrogate end point instead of an actual gain of survival [7]; and (c) we believe that many of the trials upon which these approvals were granted lack an appropriate control arm. For example, in 2019–2020, six cancer drugs have been approved by the FDA with no apparent control arm (Table 1), and in one instance despite an advisory panel’s concerns about the drug’s toxicity and the lack of randomized clinical data [4].

**Table 1:** FDA approvals in 2019–2020 for precision drugs with no control arm

Drug	Trial	Number of Patients	Comments*	References
Tazemetostat	NCT02601950	62	“*Efficacy was investigated in a single-arm cohort (Cohort 5) of a multi-center trial (Study EZH-202, NCT02601950) in patients with histologically confirmed, metastatic or locally advanced epithelioid sarcoma*.”	[8, 9]
Avapritinib	NCT02508532	43	“*Efficacy was investigated in NAVIGATOR (NCT02508532), a multi-center, single-arm, open-label trial enrolling 43 patients with GIST harboring a PDGFRA exon 18 mutation*”	[10]
Enfortumab Vedotin	NCT03219333	125	“*Efficacy was investigated in EV-201 (NCT03219333), a single-arm, multicenter trial enrolling 125 patients with locally advanced or metastatic urothelial cancer who received prior treatment with a PD-1 or PD-L1 inhibitor and platinum-based chemotherapy*.”	[11]
Zanubrutinib	NCT03206970 NCT02343120	86 32	“*Efficacy was evaluated in BGB-3111-206 (NCT03206970), a phase 2 open-label, multicenter, single-arm trial of 86 patients with MCL who received at least one prior therapy. Efficacy was also assessed in BGB-3111-AU-003 (NCT 02343120), a phase 1/2, open-label, dose-escalation, global, multicenter, single-arm trial of B‑cell malignancies, including 32 previously treated MCL patients treated with zanubrutinib administered orally at 160 mg twice daily or 320 mg once daily*.”	[12]
Entrectinib	ALKA NCT02097810 NCT02568267	54 51	“*Efficacy in NTRK-positive tumors was investigated in 54 adult patients who received entrectinib at various doses and schedules in one of three multicenter, single-arm, clinical trials: ALKA, STARTRK-1 (NCT02097810) and STARTRK-2 (NCT02568267)*” “*Efficacy in ROS1-positive metastatic NSCLC was investigated in 51 adult patients who received entrectinib at various doses and schedules in the same three trials; 90% received entrectinib 600 mg orally once daily*.”	[13–16]
Selinexor	NCT02336815	122	“Efficacy was evaluated in 122 patients enrolled in Part 2 of STORM (KCP-330-012; NCT02336815), a multicenter, single-arm, open-label study of patients with RRMM who had previously received three or more anti-myeloma treatment regimens including an alkylating agent, glucocorticoids, bortezomib, carfilzomib, lenalidomide, pomalidomide, and an anti-CD38 monoclonal antibody… …The approval was based on efficacy and safety in a prespecified subgroup analysis of 83 patients”.	[4]

*Comments Sources:

1. https://www.fda.gov/drugs/resources-information-approved-drugs/fda-approves-tazemetostat-advanced-epithelioid-sarcoma;

2. https://cacmap.fda.gov/drugs/resources-information-approved-drugs/fda-approves-avapritinib-gastrointestinal-stromal-tumor-rare-mutation;

3. https://www.fda.gov/drugs/resources-information-approved-drugs/fda-grants-accelerated-approval-enfortumab-vedotin-ejfv-metastatic-urothelial-cancer;

4. https://www.fda.gov/drugs/resources-information-approved-drugs/fda-grants-accelerated-approval-zanubrutinib-mantle-cell-lymphoma;

5. https://www.fda.gov/drugs/resources-information-approved-drugs/fda-approves-entrectinib-ntrk-solid-tumors-and-ros-1-nsclc;

6.https://www.fda.gov/drugs/resources-information-approved-drugs/fda-grants-accelerated-approval-selinexor-multiple-myeloma.

As such we believe that there is a broad consensus for the need to have unbiased patient selection (in particular with regard to age, PS, staging and including an optimal control arm) to have the necessary rigorous control for a clinical trial. Unfortunately, it is our belief that many of these simple principles are far from being homogenously applied [17, 18].

## The “Mesothelioma model”

### Immune checkpoint inhibitors

A “one-size-fits-all” approach becomes even more accepted when rare/ hard-to-treat cancers are considered as few resources are generally allocated to these cancers.

Malignant Pleural Mesothelioma (MPM), is one such rare/hard-to-treat cancer, an aggressive occupational cancer with heavy social impact, and we shall use MPM in the following sections to represent an “ideal” model to study this potential issue.

Currently we are seeing the rapid identification of potential targets and emergence of multiple novel therapies that are tested in Phase II trials for this neoplasm. All are welcome as potential as they may prove to be key turning points for the treatment of this stubborn neoplasm. On October 2, 2020 the FDA approved the use of the immune checkpoint inhibitors Ipilimumab/Nivolumab as a first-line treatment for adult patients with unresectable MPM [19], on the basis of the results from the CHECKMATE-743 (CM-743) clinical trial [20, 21].

Whilst this approval has been warmly welcomed by the clinical community as it is essentially the first major approval of a treatment of MPM in the firstline setting since the initial approval of cisplatin/pemetrexed in 2004 [22], concerns regarding the solidity and reliability of the results obtained with Immune Check-point inhibitors (ICIs) for MPM have been raised [23–26]. Indeed, using innovative statistical tools that calculate either Survival Inferred Fragility Index [SIFI], or the Restricted Mean Survival Time Difference [RMST-D] we and others have described limitations to various standard therapies for other clinical settings including ICI [27–30].

These first results, have prompted us to examine in more depth the three-year results of CM-743 and analyzed the Survival-Inferred Fragility Index (SIFI) including an additional censoring analysis of the updated three-year results of CM-743 trial [21] with the same methodology used in our previous analyses [31]. Our analysis raises some important issues. In particular we would argue that the methodology used for OS analysis and the subsequent conclusions are still associated with informative censoring [32], due to differential censoring favoring the control arm (p = 0.026) [20, 21]. It is noteworthy that, after performing a sensitivity analysis accounting for these censoring imbalances, we observe that the results were no longer significant (HR 0.85, 95%CI 0.71–1.02; p = 0.089).

Using the Survival-Inferred Fragility Index (SIFI) method with the three- year outcomes (21), we found that the SIFI was 6 patients, representing only 0.99% of the total sample size. This finding indicates that a small variation in the study population could in effect overturn the conclusions of the study, and suggest that the original trial data also lack of statistical robustness.

**Fig. 1:**
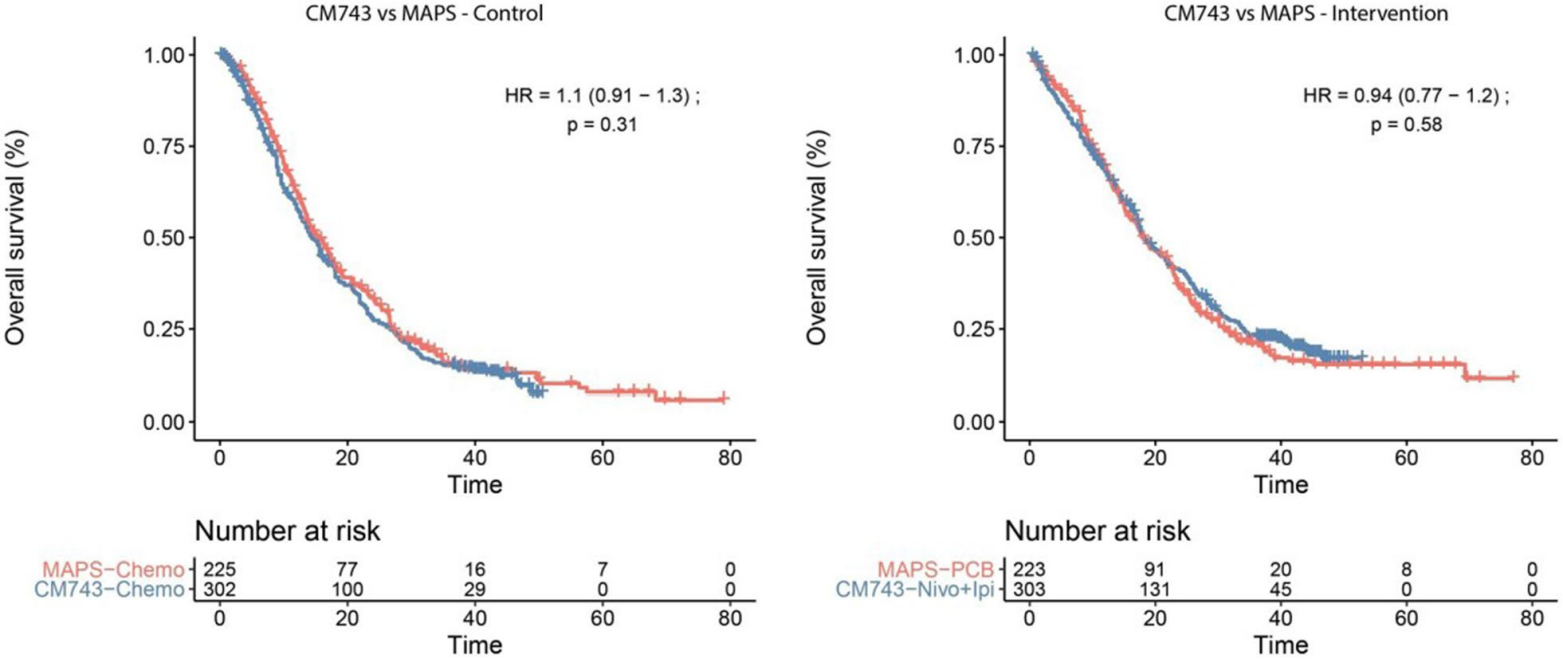
Curves show virtual comparisons between the reconstructed overall survival curves of control and intervention groups for the updated CheckMate743 and MAPS studies. HR indicates hazard ratio

In this regard, we also report that the OS curves of the interventions for the MAPS trial (33) and the updated CM-743 trial still overlap for both intervention and control (Fig. 1). It must be noted however, that such a comparison cannot be considered to be as reliable as a head-to-head comparison in a randomized control trial, nevertheless we believe it is worthy of careful consideration.

The long-term 3-year follow up data for CM-743 suggests that only non-epithelioid MPM appears to derive any benefit from Ipilimumab/Nivolumab. This by itself is a noteworthy result, as this subtype of MPM is traditionally associated with a worse outcome, and poor response to cisplatin/pemetrexed based therapy. The results of three-year-analysis of CM-743, might therefore benefit non-epithelioid histological subtype because they seem to be associated with a significant effects size.

However, we found that even in this subset severe limitations exist and, in particular, the original significant differential censoring for this subtype still represents a relevant unresolved issue that introduces a pivotal bias to the results. Alternatively, it is also possible that the observed results in the intention to treat subjects are driven by a genuine effect in the non-epithelioid subset and were masked by the lack of it in the epithelioid subtype.

There also remains the possibility that this potentially significant finding, as it may be underpowered, as underpowered studies provided with the flexibility of the exploratory analysis often produce false positive or exaggerated results [34].

Whilst compelling, we propose that the results from this subgroup analysis should be considered as hypothesis generating, because the CM-743 trial was neither designed nor powered to answer this important question. It is our belief that additional prospective and randomized trials will be required especially for the non-epithelioid -subtype, to fully establish the impact of this new therapeutic option for patients with MPM. to establish its role as a “front-line” treatment for MPM.

As far the Phase II trials of ICI published so far, the use of suboptimal control arms [35], the widespread use of surrogate primary end points [35, 36], and the concerns about patient selection (with respect to performance status 0 (PS0), young age, and very early stage) suggest that we need to revisit clinical trial design in MPM. In this regard the Phase II trial combining first line ICIs with chemotherapy [37], provides an example of this issue in that efficacy is compared to “historic” response, and roughly 50% of the study population at PS0 (ECOG PS0 of 41.8%). The validity of the benefits observed in this trial have subsequently been questioned following independent re-analysis [24].

**Fig. 2:**
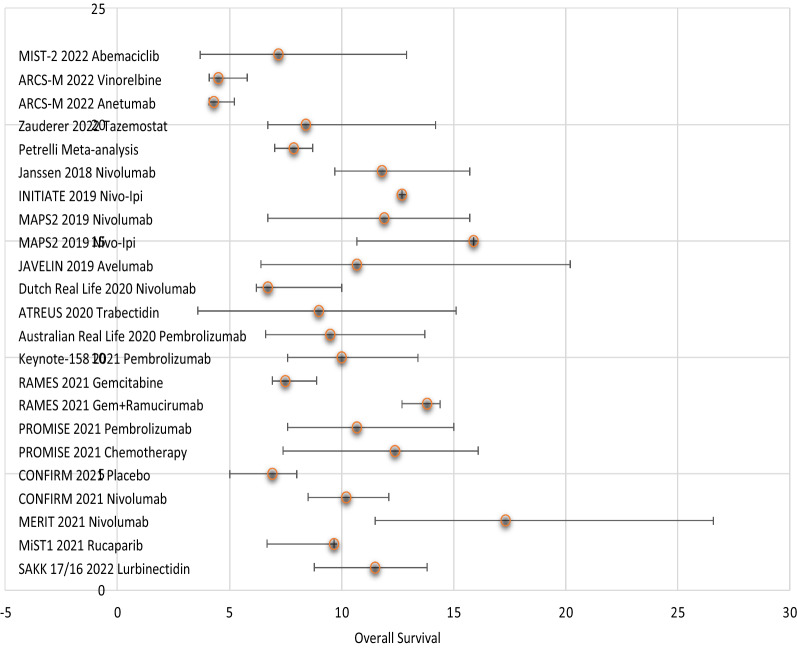
Comparison of OS achieved by the latest trial for MPM

Other authors have presumed the efficacy of Nivolumab in second line on the basis of only 34 MPM patients but not considering the very broad range of actual responses and even the broader standard deviation of the results (Fig. 2; [38]).

### New targeted therapies

The issues with regard to inappropriate trial design/methodology are not restricted to those of just ICI. Lack of or suboptimal control arms have been raised frequently as an issue in several of the recent Phase II trials of new agents/therapies in MPM [39–41].

### The current problem with biomarkers

Many of the current clinical trials conducted in MPM have utilized a basket case approach, often involving the use of candidate biomarkers [42]. However, in many instances the pre-clinical data predicating these biomarkers may be flawed. In the following sections we shall discuss in more depth some additional examples of Phase II trials that raise concern. All center on a gene whose expression is frequently inactivated/lost in MPM, BRCA1-associated Protein 1 (BAP1) [43], or to BRCA1 itself.

It was first suggested that BAP1 inactivation/loss was associated with sensitivity to enhancer of zeste 2 (EZH2) inhibitors in MPM (44). However, a Phase II trial of an EZH2 inhibitor (Tezemetostat) has been completed in MPM [45]. In this trial the authors state that despite stratifying the responders by BAP1 status no statistically significant differences (p = 0.264) in survival were observed indicating BAP1 mutational status has no influence on response to Tezemetostat [45].

In a similar manner, BAP1 inactivation/mutation was linked to sensitivity to Poly(ADP-Ribose) Polymerase (PARP) inhibitors [46, 47]. Two Phase II clinical trials of PARPi in MPM have now been conducted which have assessed whether BAP1 status plays a role in sensitivity/response [48, 49]. In both trials, overall it can be concluded that PARPi have limited activity in MPM including patients with BAP1 mutations, further confirmed by an independent study [50], and the rationale for the supposed efficacy of PARP1 inhibitors in MPM patients bearing BAP1 mutation remains inconclusive [48, 51, 52], and warrants further attention.

### BRCA1, BAP1 and vinorelbine

A potential role for BRCA1 as a biomarker for sensitivity to vinorelbine was identified in 2012 [53], and potentially confirmed through a pooled analysis [54]. On the basis of this observation a Phase II clinical trial (NCT02139904) was conducted with patients randomized 2:1 to receive either active symptom control with oral vinorelbine versus active symptom control (ASC) every 3 weeks until disease progression, unacceptable toxicity or withdrawal [55], and whilst the trial met its stated primary goal with respect to improved PFS, BRCA1 did not predict resistance to ASC + vinorelbine [55]. This appears to confirm the earlier observation by others that BRCA1 was not a good biomarker for stratifying sensitivity to vinorelbine/cisplatin treatment in MPM [56].

Intriguingly, an analysis of some of the patients treated with vinorelbine demonstrated that loss of BRCA1 or that of a separate new biomarker MAD2L1, a gene transcriptionally regulated by BRCA1 [57]. Moreover, a retrospective analysis of the MS01 trial (NCT00075699), found a small, though non-significant, overall survival disadvantage associated with BAP1 expression in tumors from patients treated with vinorelbine (58). These results suggest that a more comprehensive Phase III biomarker driven trial is warranted in order to truly determine the potential utility of BAP1, BRCA1 and MAD2L1 as biomarkers for sensitivity to vinorelbine/cisplatin combinations.

Returning to PARPi, it must be noted that a Phase II study examining the combination of Niraparib plus Dostarlimab is currently being conducted (NCT04940637) in NCSLC and MPM [59], for patients with confirmed positivity for germline or somatic homologous recombination deficient (HRD) status and tumor PD-L1 expression (tumor proportion score 1%) and must have experienced disease progression or recurrence during or after at least 1 systemic therapy for advanced metastatic disease. Unfortunately, the study suffers from some of the weaknesses that we have already raised for other trial methodologies such as ECOG status 0 and is limited by being a single-arm prospective study, with the outcome design generated on assumptions for PFS based on historical data.

This problem is compounded by the number of new second-line treatments tested so far, as none appear to top the survival of the drugs currently used in the same settings (Fig. 2) and that in general ICIs for MPM do not show any true superiority compared to standard treatments within real world settings [23, 60], a matter that we have raised with respect to the results of several other trials for MPM recently published [35, 39, 40].

### The financial impact: are we bearing the brunt?

Previously in 2011, the Oncology Commission of Lancet stated: “. . the cancer profession and industry should take responsibility and not accept a sub-standard evidence base and an ethos of very small benefit at whatever cost; rather, we need delivery of fair process and a real value from new technologies”, to achieve reliable and quantitative evaluations of health outcomes and costs, for both equity and affordability [61].

**Fig. 3:**
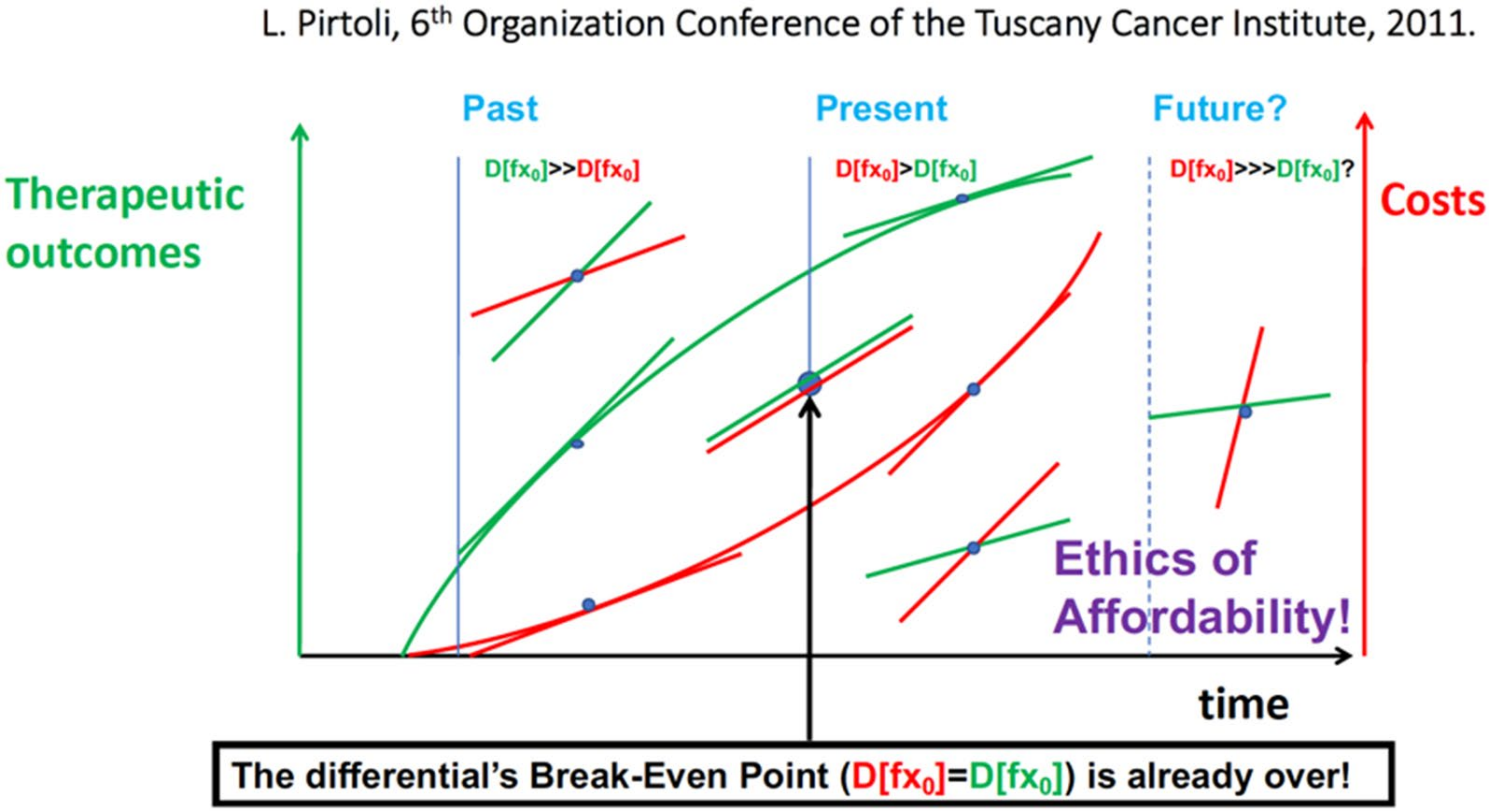
The rate of growth in costs (red) vs. outcomes (green) of cancer care, is here expressed as the derivative D[fx_0_] of both functions (i.e.: the angular coefficient at any specific point of each field)

Since then, numerous attempts have been made to draw attention to the medical and scientific communities that, whilst these new developments in cancer treatment have emerged, with outcomes that have benefited patients justifying their high costs costs, currently, this benefit/cost ratio has progressively reversed up to a break-even point and we are now approaching a situation of unaffordability of cancer care, and to our mind as a priority, this trend imposes a challenge regarding the ethics of affordability for future cancer treatment. (Fig. 3) [62]. Our results fit with recent analyses which have determined that in particular, ICI combinations such as Ipi/Nivo exceeds the willingness-to-pay threshold from the perspective of US payers for the treatment of MPM [63–65]., which place the costing for the incremental cost-effectiveness ratio (ICER) of $372,414.28/QALY [64] and ICER of $475,677/QALY [63] for the newly approved ICI combination in MPM respectively. In this regard, the Phase III CONFIRM trial [66] has been cost-effectiveness built into its trial design and should provide some important data in this regard.

### How can we improve on this?

To put this into perspective, a recent analysis suggested that the approximate cost for a 24-week treatment of vinorelbine is $515, while that for six cycles of gemcitabine is approximately $887.76 [67], and the cost-effectiveness for the standard cisplatin/pemetrexed using QALY in the UK was ranges from £20,475 to £68,598 between mean and median survival [68], whilst the cost-effectiveness (ICER) of adding bevacizumab to this regimen was estimated at $727,202.589 per QALY [69] (which not unnaturally has led to its poor uptake as a first-line combination therapy in MPM.

Given the low costs associated with the use of vinorelbine and gemcitabine, can we improve of their potential utility in the clinic for the treatment of MPM? In this regard, the use of biomarkers will become imperative. In this regard, 

BAP1 status and sensitivity to cisplatin/pemetrexed, and gemcitabine, coupled with OS has recently been identified as a potentially key biomarker to identify patients that could benefit from this drug [70, 71], or to stratify patients away from cisplatin based therapeutic regimens [72], and to also potentially stratify them for vinorelbine sensitivity (albeit alongside the additional use of BRCA1 and MAD2L1).

Clearly, the use of biomarker based clinical trials such as MIST1 and MIST2 [49, 73], provide a suggestive methodology to achieve these, but the underlying pre-clinical data, patients selection, power and appropriate statistical approaches will be required. However, such basket-case or umbrella also comes with significant ethical challenges [74], and their results come with the real risk of over hype [75]. In real-terms, such an approach may require a large-scale Phase III clinical trial, with to truly determine efficacy.

## Conclusion

The current state of affairs with respect to the latest clinical trials has led to excitement in the field and suggestions that “…*we have finally turned the corner in our battle against this devastating disease*…” [76]. We believe that the treatment options for MPM have not yet been achieved, in part as their superiority versus current standard-of-care is taken for granted despite to our minds a lack of solid evidence. We think that these sort of statements are not only baseless but can also generate hopes and expectations not underpinned by facts [26, 31, 76–78].

Moreover, concerns have recently been raised with respect to both the design and conduction of clinical cancer trials which imply that flawed study designs are a central issue of concern [79]. In addition, the over-hype of such flawed trials can result in pressure on health care systems to provide these expensive agents [79].

Therefore, after many years, unfortunately we conclude that more than ever we need additional pre-clinical screening of any new proposed cancer treatment, as well as a greater methodological rigor in trial statistics, and cost/effective ratio analysis to ensure the best efficacy to our patients and sustainability to Health Systems.

## Data Availability

Not applicable.

## References

[1] Correale P, Pentimalli F, Baglio G, Krstic-Demonacos M, Saladino RE, Giordano A (2021). Is there already a need of reckoning on cancer immunotherapy?. Front Pharmacol.

[2] Chow RD, Bradley EH, Gross CP (2022). Comparison of cancer-related spending and mortality rates in the US vs 21 high-income countries. JAMA Health Forum.

[3] Zhang Y, Naci H, Wagner AK, Xu Z, Yang Y, Zhu J (2022). Overall survival benefits of cancer drugs approved in China from 2005 to 2020. JAMA Netw Open.

[4] XPO1 Inhibitor Approved for Multiple Myeloma. Cancer Discov. 2019;9(9):1150-1151. 10.1158/2159-8290.CD-NB2019-085. Epub 2019 Jul 22. PMID: 31332020. 10.1158/2159-8290.CD-NB2019-085 31332020

[5] DasGupta R, Yap A, Yaqing EY, Chia S (2022). Evolution of precision oncology-guided treatment paradigms. WIREs
Mech Dis.

[6] Olivier T, Haslam A, Prasad V (2021). Anticancer drugs approved by the US food and drug administration from 2009 to 2020 according to their mechanism of action. JAMA Netw Open.

[7] Vincent Rajkumar S (2020). The high cost of prescription drugs: causes and solutions. Blood Cancer J.

[8] Stacchiotti S, Schoffski P, Jones R, Agulnik M, Villalobos VM, Jahan TM (2019). Safety and efficacy of tazemetostat, a first-in-class EZH2 inhibitor, in patients (pts) with epithelioid sarcoma (ES) (NCT02601950). J Clin Oncol.

[9] Hoy SM (2020). Tazemetostat. First Approval Drugs.

[10] Avapritinib Approved for GIST Subgroup. Cancer Discov. 2020;10(3):334. 10.1158/2159-8290.CDNB2020-002 (Epub 2020 Jan 23. PMID: 31974168). 10.1158/2159-8290.CD-NB2020-002 31974168

[11] Rosenberg JE, O’Donnell PH, Balar AV, McGregor BA, Heath EI, Yu EY (2019). Pivotal trial of enfortumab vedotin in urothelial carcinoma after platinum and anti-programmed death 1/programmed death ligand 1 therapy. J Clin Oncol.

[12] Li G, Liu X, Chen X (2020). Simultaneous development of zanubrutinib in the USA and China. Nat Rev Clin Oncol.

[13] Doebele RC, Drilon A, Paz-Ares L, Siena S, Shaw AT, Farago AF (2020). Entrectinib in patients with advanced or metastatic NTRK fusion-positive solid tumours: integrated analysis of three phase 1–2 trials. Lancet Oncol.

[14] Marcus L, Donoghue M, Aungst S, Myers CE, Helms WS, Shen G (2021). FDA approval summary: entrectinib for the treatment of NTRK gene fusion solid tumors. Clin Cancer Res.

[15] Drilon A, Siena S, Dziadziuszko R, Barlesi F, Krebs MG, Shaw AT (2020). Entrectinib in ROS1 fusion-positive non-small-cell lung cancer: integrated analysis of three phase 1–2 trials. Lancet Oncol.

[16] Sartore-Bianchi A, Pizzutilo EG, Marrapese G, Tosi F, Cerea G, Siena S (2020). Entrectinib for the treatment of metastatic NSCLC: safety and efficacy. Expert Rev Anticancer Ther.

[17] Shen C, Ferro EG, Xu H, Kramer DB, Patell R, Kazi DS (2021). Underperformance of contemporary phase III oncology trials and strategies for improvement. J Natl Compr Canc Netw.

[18] Hahn AW, Dizman N, Msaouel P (2022). Missing the trees for the forest: most subgroup analyses using forest plots at the ASCO annual meeting are inconclusive. Ther Adv Med Oncol..

[19] Nakajima EC, Vellanki PJ, Larkins E, Chatterjee S, Mishra-Kalyani PS, Bi Y (2022). FDA approval summary: nivolumab in combination with ipilimumab for the treatment of unresectable malignant pleural mesothelioma. Clin Cancer Res.

[20] Baas P, Scherpereel A, Nowak AK, Fujimoto N, Peters S, Tsao AS (2021). First-line nivolumab plus ipilimumab in unresectable malignant pleural mesothelioma (CheckMate 743): a multicentre, randomised, open-label, phase 3 trial. Lancet.

[21] Peters S, Scherpereel A, Cornelissen R, Oulkhouir Y, Greillier L, Kaplan MA (2022). First-line nivolumab plus ipilimumab versus chemotherapy in patients with unresectable malignant pleural mesothelioma: 3-year outcomes from CheckMate 743. Ann Oncol.

[22] Hazarika M, White RM, Johnson JR, Pazdur R (2004). FDA drug approval summaries: pemetrexed (Alimta). Oncologist.

[23] Kerrigan K, Jo Y, Chipman J, Haaland B, Puri S, Akerley W (2022). A real-world analysis of the use of systemic therapy in malignant pleural mesothelioma and the differential impacts on overall survival by practice pattern. JTO Clin Res Rep.

[24] Messori A, Trippoli S (2022). Current treatments for inoperable mesothelioma: indirect comparisons based on individual patient data reconstructed retrospectively from 4 trials. J Chemother.

[25] Tagliamento M, Bironzo P, Curcio H, De Luca E, Pignataro D, Rapetti SG (2022). A systematic review and meta-analysis of trials assessing PD-1/PD-L1 immune checkpoint inhibitors activity in pre-treated advanced stage malignant mesothelioma. Crit Rev Oncol Hematol.

[26] Pass HI. Commentary: A chess game for mesothelioma treatment: Not checkmate yet! J Thorac Cardiovasc Surg. 2022;S0022-5223(22)00127-1. 10.1016/j.jtcvs.2022.02.003 (Epub ahead of print. PMID: 35227497). 10.1016/j.jtcvs.2022.02.003 35227497

[27] Bomze D, Asher N, Hasan Ali O, Flatz L, Azoulay D, Markel G (2020). Survival-inferred fragility index of phase 3 clinical trials evaluating immune checkpoint inhibitors. JAMA Netw Open.

[28] Bomze D, Azoulay D, Meirson T (2020). Immunotherapy with programmed cell death 1 vs programmed cell death ligand 1 inhibitors in patients with cancer. JAMA Oncol.

[29] Pak K, Uno H, Kim DH, Tian L, Kane RC, Takeuchi M (2017). Interpretability of cancer clinical trial results using restricted mean survival time as an alternative to the hazard ratio. JAMA Oncol.

[30] Gilboa S, Pras Y, Mataraso A, Bomze D, Markel G, Meirson T (2021). Informative censoring of surrogate end-point data in phase 3 oncology trials. Eur J Cancer.

[31] Meirson T, Pentimalli F, Cerza F, Baglio G, Gray SG, Correale P (2022). Comparison of 3 randomized clinical trials of frontline therapies for malignant pleural mesothelioma. JAMA Netw Open.

[32] Templeton AJ, Amir E, Tannock IF (2020). Informative censoring—a neglected cause of bias in oncology trials. Nat Rev Clin Oncol.

[33] Zalcman G, Mazieres J, Margery J, Greillier L, Audigier-Valette C, Moro-Sibilot D (2016). Bevacizumab for newly diagnosed pleural mesothelioma in the Mesothelioma Avastin Cisplatin Pemetrexed Study (MAPS): a randomised, controlled, open-label, phase 3 trial. Lancet.

[34] Nord CL, Valton V, Wood J, Roiser JP (2017). Power-up: a reanalysis of ‘power failure’ in neuroscience using mixture modeling. J Neurosci.

[35] Correale P, Pentimalli F, Nardone V, Giordano A, Mutti L (2022). CONFIRM trial: what is the real efficacy of second-line immunotherapy in mesothelioma?. Lancet Oncol.

[36] Calabrò L, Rossi G, Morra A, Rosati C, Cutaia O, Daffinà MG (2021). Tremelimumab plus durvalumab retreatment and 4-year outcomes in patients with mesothelioma: a follow-up of the open label, non-randomised, phase 2 NIBIT-MESO-1 study. Lancet Respir Med.

[37] Forde PM, Anagnostou V, Sun Z, Dahlberg SE, Kindler HL, Niknafs N (2021). Durvalumab with platinum-pemetrexed for unresectable pleural mesothelioma: survival, genomic and immunologic analyses from the phase 2 PrE0505 trial. Nat Med.

[38] Fujimoto N, Okada M, Kijima T, Aoe K, Kato T, Nakagawa K (2021). Clinical efficacy and safety of nivolumab in Japanese patients with malignant pleural mesothelioma: 3-year results of the MERIT study. JTO Clin Res Rep.

[39] Barbarino M, Bottaro M, Luzzi L, Giordano A, Mutti L (2020). Tumour treating fields for mesothelioma. Lancet Oncol.

[40] Porta C, Nardone V, Gray SG, Correale P, Mutti L (2021). RAMES study: is there really a role for VEGF inhibition in mesothelioma?. Lancet Oncol.

[41] Nardone V, Porta C, Giannicola R, Correale P, Mutti L (2022). Abemaciclib for malignant pleural mesothelioma. Lancet Oncol.

[42] Zabor EC, Kane MJ, Roychoudhury S, Nie L, Hobbs BP (2022). Bayesian basket trial design with false-discovery rate control. Clin Trials.

[43] Carbone M, Harbour JW, Brugarolas J, Bononi A, Pagano I, Dey A (2020). Biological mechanisms and clinical significance of BAP1 mutations in human cancer. Cancer Discov.

[44] LaFave LM, Béguelin W, Koche R, Teater M, Spitzer B, Chramiec A (2015). Loss of BAP1 function leads to EZH2-dependent transformation. Nat Med.

[45] Zauderer MG, Szlosarek PW, Le Moulec S, Popat S, Taylor P, Planchard D (2022). EZH2 inhibitor tazemetostat in patients with relapsed or refractory, BAP1-inactivated malignant pleural mesothelioma: a multicentre, open-label, phase 2 study. Lancet Oncol.

[46] Borchert S, Wessolly M, Schmeller J, Mairinger E, Kollmeier J, Hager T (2019). Gene expression profiling of homologous recombination repair pathway indicates susceptibility for olaparib treatment in malignant pleural mesothelioma in vitro. BMC Cancer.

[47] Parrotta R, Okonska A, Ronner M, Weder W, Stahel R, Penengo L (2017). A Novel BRCA1-associated protein-1 isoform affects response of mesothelioma cells to drugs impairing BRCA1-mediated DNA repair. J Thorac Oncol.

[48] Ghafoor A, Mian I, Wagner C, Mallory Y, Agra MG, Morrow B (2021). Phase 2 study of olaparib in malignant mesothelioma and correlation of efficacy with germline or somatic mutations in BAP1 gene. JTO Clin Res Rep.

[49] Fennell DA, King A, Mohammed S, Branson A, Brookes C, Darlison L (2021). Rucaparib in patients with BAP1-deficient or BRCA1-deficient mesothelioma (MiST1): an open-label, single-arm, phase 2a clinical trial. Lancet Respir Med.

[50] Dudnik E, Bar J, Moore A, Gottfried T, Moskovitz M, Dudnik J (2021). BAP1-altered malignant pleural mesothelioma: outcomes with chemotherapy, immune check-point inhibitors and poly(ADP-Ribose) polymerase inhibitors. Front Oncol.

[51] Pinton G, Manente AG, Murer B, De Marino E, Mutti L, Moro L (2013). PARP1 inhibition affects pleural mesothelioma cell viability and uncouples AKT/mTOR axis via SIRT1. J Cell Mol Med.

[52] Gabano E, Pinton G, Balzano C, Boumya S, Osella D, Moro L (2021). Unsymmetric cisplatin-based Pt(IV) conjugates containing a PARP-1 inhibitor pharmacophore tested on malignant pleural mesothelioma cell lines. Molecules..

[53] Busacca S, Sheaff M, Arthur K, Gray SG, O’Byrne KJ, Richard DJ (2012). BRCA1 is an essential mediator of vinorelbine-induced apoptosis in mesothelioma. J Pathol.

[54] He Q, Zhang M, Zhang J, Zhong S, Liu Y, Shen J (2016). Predictive value of BRCA1 expression on the efficacy of chemotherapy based on anti-microtubule agents: a pooled analysis across different malignancies and agents. Ann Transl Med.

[55] Fennell DA, Porter C, Lester J, Danson S, Taylor P, Sheaff M (2022). Active symptom control with or without oral vinorelbine in patients with relapsed malignant pleural mesothelioma (VIM): a randomised, phase 2 trial. EClinicalMedicine.

[56] Zimling ZG, Sørensen JB, Gerds TA, Bech C, Andersen CB, Santoni-Rugiu E (2012). A biomarker profile for predicting efficacy of cisplatin-vinorelbine therapy in malignant pleural mesothelioma. Cancer Chemother Pharmacol.

[57] Busacca S, O’Regan L, Singh A, Sharkey AJ, Dawson AG, Dzialo J (2021). BRCA1/MAD2L1 deficiency disrupts the spindle assembly checkpoint to confer vinorelbine resistance in mesothelioma. Mol Cancer Ther.

[58] Kumar N, Alrifai D, Kolluri KK, Sage EK, Ishii Y, Guppy N (2019). Retrospective response analysis of BAP1 expression to predict the clinical activity of systemic cytotoxic chemotherapy in mesothelioma. Lung Cancer.

[59] Passiglia F, Bironzo P, Righi L, Listì A, Arizio F, Novello S (2021). A prospective phase II single-arm study of niraparib plus dostarlimab in patients with advanced non-small-cell lung cancer and/or malignant pleural mesothelioma, positive for PD-L1 expression and germline or somatic mutations in the DNA repair genes: rationale and study design. Clin Lung Cancer.

[60] Cedres S, Assaf JD, Iranzo P, Callejo A, Pardo N, Navarro A (2021). Efficacy of chemotherapy for malignant pleural mesothelioma according to histology in a real-world cohort. Sci Rep.

[61] Sullivan R, Peppercorn J, Sikora K, Zalcberg J, Meropol NJ, Amir E (2011). Delivering affordable cancer care in high-income countries. Lancet Oncol.

[62] Pirtoli L, Alia L, Zacchini S (2021). Oncology and a time of crisis. Science, complexity, ethic values, and incertitude. An argumentative essay. Medicus.

[63] Yang L, Cao X, Li N, Zheng B, Liu M, Cai H (2022). Cost-effectiveness analysis of nivolumab plus ipilimumab versus chemotherapy as the first-line treatment for unresectable malignant pleural mesothelioma. Ther Adv Med Oncol.

[64] Ye ZM, Tang ZQ, Xu Z, Zhou Q, Li H (2022). Cost-effectiveness of nivolumab plus ipilimumab as first-line treatment for American patients with unresectable malignant pleural mesothelioma. Front Public Health.

[65] Michaeli DT, Michaeli T. Overall Survival, Progression-free survival, and tumor response benefit supporting initial US food and drug administration approval and indication extension of new cancer drugs, 2003-2021. J Clin Oncol. 2022;JCO2200535. 10.1200/JCO.22.00535 (Epub ahead of print. PMID: 35921606) 10.1200/JCO.22.00535 35921606

[66] Fennell DA, Kirkpatrick E, Cozens K, Nye M, Lester J, Hanna G (2018). CONFIRM: a double-blind, placebo-controlled phase III clinical trial investigating the effect of nivolumab in patients with relapsed mesothelioma: study protocol for a randomised controlled trial. Trials.

[67] Borrelli EP, McGladrigan CG (2021). A review of pharmacologic management in the treatment of mesothelioma. Curr Treat Options Oncol.

[68] Cordony A, Le Reun C, Smala A, Symanowski JT, Watkins J (2008). Cost-effectiveness of pemetrexed plus cisplatin: malignant pleural mesothelioma treatment in UK clinical practice. Value Health.

[69] Zhan M, Zheng H, Xu T, Yang Y, Li Q (2017). Cost-effectiveness analysis of additional bevacizumab to pemetrexed plus cisplatin for malignant pleural mesothelioma based on the MAPS trial. Lung Cancer.

[70] Louw A, Panou V, Szejniuk WM, Meristoudis C, Chai SM, van Vliet C (2022). BAP1 loss by immunohistochemistry predicts improved survival to first-line platinum and pemetrexed chemotherapy for patients with pleural mesothelioma: a validation study. J Thorac Oncol.

[71] Guazzelli A, Meysami P, Bakker E, Demonacos C, Giordano A, Krstic-Demonacos M (2019). BAP1 status determines the sensitivity of malignant mesothelioma cells to gemcitabine treatment. Int J Mol Sci..

[72] Oehl K, Vrugt B, Wagner U, Kirschner MB, Meerang M, Weder W (2021). Alterations in BAP1 are associated with cisplatin resistance through inhibition of apoptosis in malignant pleural mesothelioma. Clin Cancer Res.

[73] Fennell DA, King A, Mohammed S, Greystoke A, Anthony S, Poile C (2022). Abemaciclib in patients with p16ink4A-deficient mesothelioma (MiST2): a single-arm, open-label, phase 2 trial. Lancet Oncol.

[74] Strzebonska K, Waligora M (2019). Umbrella and basket trials in oncology: ethical challenges. BMC Med Ethics.

[75] Janiaud P, Serghiou S, Ioannidis JPA (2019). New clinical trial designs in the era of precision medicine: an overview of definitions, strengths, weaknesses, and current use in oncology. Cancer Treat Rev.

[76] Kindler HL (2022). Systemic therapy for mesothelioma: turning the corner. JCO Oncol Pract.

[77] Nowak AK, Jackson A, Sidhu C (2022). Management of advanced pleural mesothelioma-at the crossroads. JCO Oncol Pract.

[78] Fennell DA, Dulloo S, Harber J (2022). Immunotherapy approaches for malignant pleural mesothelioma. Nat Rev Clin Oncol.

[79] Ratain MJ (2022). Oncology drug prescribing: the influences of greed and fear. JCO Oncol Pract.

